# Protein delivery in intermittent and continuous enteral nutrition with a protein-rich formula in critically ill patients—a protocol for the prospective randomized controlled proof-of-concept Protein Bolus Nutrition (Pro BoNo) study

**DOI:** 10.1186/s13063-020-04635-1

**Published:** 2020-08-25

**Authors:** Simona Reinhold, Desirée Yeginsoy, Alexa Hollinger, Atanas Todorov, Lionel Tintignac, Michael Sinnreich, Caroline Kiss, Caroline E. Gebhard, Balázs Kovács, Bianca Gysi, Lara Imwinkelried, Martin Siegemund

**Affiliations:** 1 Intensive Care Unit, University Hospital Basel, Basel, Switzerland; 2 Department of Biomedicine, University Hospital Basel, Basel, Switzerland; 3 Departments of Biomedicine and Neurology, Centre of Neuromuscular Diseases, University Hospital Basel, Basel, Switzerland; 4 Department of Clinical Nutrition, University Department of Geriatric Medicine Felix Platter, Basel, Switzerland; 5 Department of Radiology, Musculoskeletal Diagnostics, University Hospital Basel, Basel, Switzerland

**Keywords:** Nutrition and dietetics, Adult intensive and critical care, Ultrasound

## Abstract

**Background:**

Critically ill patients rapidly develop muscle wasting resulting in sarcopenia, long-term disability and higher mortality. Bolus nutrition (30–60 min period), whilst having a similar incidence of aspiration as continuous feeding, seems to provide metabolic benefits through increased muscle protein synthesis due to higher leucine peaks.

To date, clinical evidence on achievement of nutritional goals and influence of bolus nutrition on skeletal muscle metabolism in ICU patients is lacking. The aim of the Pro BoNo study (Protein Bolus Nutrition) is to compare intermittent and continuous enteral feeding with a specific high-protein formula. We hypothesise that target quantity of protein is reached earlier (within 36 h) by an intermittent feeding protocol with a favourable influence on muscle protein synthesis.

**Methods:**

Pro BoNo is a prospective randomised controlled study aiming to compare the impact of intermittent and continuous enteral feeding on preventing muscle wasting in 60 critically ill patients recruited during the first 48 h after ICU admission. The primary outcome measure is the time until the daily protein target (≥ 1.5 g protein/kg bodyweight/24 h) is achieved. Secondary outcome measures include tolerance of enteral feeding and evolution of glucose, urea and IGF-1. Ultrasound and muscle biopsy of the quadriceps will be performed.

**Discussion:**

The Basel Pro BoNo study aims to collect innovative data on the effect of intermittent enteral feeding of critically ill patients on muscle wasting.

**Trial registration:**

ClinicalTrials.gov NCT03587870. Registered on July 16, 2018. Swiss National Clinical Trials Portal SNCTP000003234. Last updated on July 24, 2019.

## Administrative information

**Table:** 

Title	Protein delivery in intermittent and continuous enteral nutrition with a protein-rich formula in critically ill patients A protocol for the prospective randomised controlled proof-of-concept Protein Bolus Nutrition (Pro BoNo) study
Trial registration	ClinicalTrials.gov Identifier: NCT03587870, registered July 16, 2018. Swiss National Clinical Trial Portal identifier: SNCTP000003234, last updated July 24, 2019.
Protocol version	Clinical Study Protocol Version 4, 15.07.2019
Funding	Goldschmidt-Jacobson Foundation (approval June 2018): full salary for one doctoral student working full time on this study
Author details	Simona Reinhold^1^*, Desirée Yeginsoy^1^*, Alexa Hollinger^1^, Atanas Todorov^1^, Lionel Tintignac^2^, Michael Sinnreich^3^, Caroline Kiss^4^, Caroline E. Gebhard^1^, Balázs Kovács^5^, Bianca Gysi^1^, Lara Imwinkelried^1^, Martin Siegemund^1^ ^1^Intensive Care Unit, University Hospital Basel, Basel, Switzerland ^2^Department of Biomedicine, University Hospital Basel, Basel, Switzerland ^3^Departments of Biomedicine and Neurology, Centre of Neuromuscular Diseases, University Hospital Basel, Basel, Switzerland ^4^Department of Clinical Nutrition, University Department of Geriatric Medicine Felix Platter, Basel, Switzerland ^5^Department of Radiology, Musculoskeletal Diagnostics, University Hospital Basel, Basel, Switzerland
Name and contact information for the trial sponsor	Martin Siegemund, MD Deputy Head of Intensive Care Unit University Hospital Basel Tel. +41 61 328 64 14
Role of sponsor	Concept and design of study. Coordination of study together with the Intensive Care Unit research team of the University Hospital Basel. Responsibility for the integrity of the data and the accuracy of the data analysis.

## Strengths and limitations of this study

- The study’s main strength is the implementation of an advanced and promising therapy for an unsolved problem: rapid muscle wasting in critically ill patients. The short intermittent feeding times in the experimental group (a total of 2 h feeding time and total break time of 22 h) may allow more flexible patient care compared to the continuous control group (a total of 20 h feeding time and total break time of 4 h).
- If the short intermittent feeding times are well tolerated, patients in the experimental group are expected to reach the daily protein target faster, as there are fewer feeding interruptions.
- Until today, there is no known study that directly measures muscle atrophy by ultrasound and biopsy in relation to differing feeding protocols in critically ill patients. Due to the quantitative measurement of the quadriceps muscle by ultrasound and histological evaluation of biopsies, the study could provide new reliable data in the field of muscle wasting in critically ill patients and a direct comparison of the two feeding regimens.
- The study is limited by the wide heterogeneity of type and severity of underlying disease of intensive care unit (ICU) patients included in the study. These variable conditions will be addressed according to assessment and analysis of the Simplified Acute Physiology Score II (SAPS II) score.

## Introduction

### Background and rationale

Acute skeletal muscle degradation in intensive care unit (ICU) patients is associated with functional impairment and increased short- and long-term morbidity [1, 2]. Muscle weakness in ICU patients occurs early and rapidly due to stress metabolism, immobility and malnutrition [3, 4]. Physical disability can persist up to 5 years [5], or possibly even longer. The loss of muscle mass seems to be accented during the first 2 to 3 weeks of immobilisation [6]. Moreover, malnutrition represents an important factor contributing to muscle wasting [7]. Therefore, adequate nutritional support could contribute to diminish muscle wasting in ICU patients.

Oral intake of 20 g protein per meal for healthy, respectively 30 g protein for the elderly, is needed for adequate muscle protein synthesis (MPS) [8]. When comparing the two main components of milk protein casein and whey protein, the latter has gained more and more importance in MPS [9]. Whey protein is digested faster and stimulates postprandial amino acid accretion more effectively [9–11]. Regarding MPS, the vital role of the essential amino acid leucine, naturally available in relevant amounts when administering whey protein (1.13 g leucine/100 ml) [12], has been described in prior investigations [10, 13, 14]. In 2015, Marik [1] suggested feeding whey-based nutrition formulas to critically ill patients, and in 2017, Heyland et al. [15] proposed aiming for high-protein nutrition targets (minimum 1.2–1.5 g protein/kg bodyweight (BW)/24 h, maximum 2.0–2.5 g protein/kg BW/24 h) in ICU setting. To the best of our knowledge, to date, no trial investigating the impact of high-protein whey-based formulas on muscle state of critically ill patients has been performed.

The administration of intermittent feeding (IF) versus continuous feeding (CF) is a topic of ongoing controversy [1, 16–24]. The possible benefits of IF have overcome concerns of adverse effects such as pulmonary aspiration and have blazed a trail for further investigations challenging the definition of continuous feeding as the standard for ICU patients [21, 25]. Insulin plays an essential role in MPS in the presence of amino acids and importantly blunts muscle protein breakdown [26]. Earlier investigations reported that MPS could only be stimulated for a limited time in healthy individuals, despite sufficient availability of amino acids [27]. Another study in healthy men revealed that plasma insulin concentrations increased significantly to a maximum of + 303% for 60 min after application of an oral protein bolus and then decreased after 90 min [8]. Moreover, in 2015, Hooper and Marik suggested in their reviews based on the findings of trials in humans and animals that enteral nutrition given continuously would even reduce protein synthesis [1, 11].

In addition, compared to continuous feeding, IF is assumed to have direct potential advantages over gastrointestinal-hormone secretion [21, 28] and indirect advantages due to preservation of pancreatic and gallbladder function [21], elevated insulin secretion [28, 29] and improvement of glucose control [1]. However, whether this physiologic hormone pattern translates into molecular muscle anabolism remains unknown.

In this study, we hypothesise that target quantity of protein is reached earlier (within 36 h) with enteral IF compared to standard CF. In addition, we expect IF to be equally well tolerated, incidence of adverse events to be similar between groups and MPS to be higher in the experimental group.

### Objectives

The Pro BoNo study (Protein Bolus Nutrition) combines administration of a high-protein whey-based enteral formula with IF to investigate the effects on MPS and muscle rebuilding in ICU patients by muscle biopsy [8, 25, 30] and ultrasound [31–33]. CF with the same formula will be performed in the control group. The potential of IF to reach nutritional goals earlier will be assessed. In addition, adverse effects of IF such as gastrointestinal symptoms, metabolic parameters (blood glucose) and aspiration will be analysed.

For information on overall risk of mortality, organ dysfunction, disease severity and nutritional state of studied patients, standardised scores will be utilised.

### Trial design

The Basel Pro BoNo is a prospective randomised controlled unblinded, investigator-initiated, single-centre study in critically ill patients expected to require nutritional support on the ICU for at least 5 days.

## Methods: participants, interventions and outcomes

### Study setting

Patients will be observed for a maximum of 7 days during their stay in an adult 50-bed ICU admitting medical or surgical patients.

### Eligibility criteria

#### Study population

##### Inclusion criteria

Participants fulfilling the following criteria will be eligible for inclusion in the study:

- Inclusion within the first 48 h after ICU admission
- Adult patients (aged ≥ 18 years)
- Expected to receive enteral feeding for at least 5 days
- Expected ICU stay of at least 5 days

##### Exclusion criteria

Participants meeting the following criteria will be excluded from the study:

- Start of enteral nutrition before ICU admission or study inclusion
- Pregnant or breast feeding women (aged ≤ 45 years will be tested for beta-human chorionic gonadotropin in urine or serum)
- Clinically significant acute or chronic kidney failure with a glomerular filtration rate (GFR) < 20 ml/min
- Body mass index (BMI) ≤ 18 or ≥ 35 kg/m^2^
- Intestinal perforation, peritonitis, intestinal fistula, necrosis or other contraindication to enteral nutrition
- Noradrenaline ≥ 0.5 μg/kg bodyweight/min
- Inherited/chronic skeletal muscle disorder (e.g. motor neuron disease)
- Paralysis (e.g. hemiplegia, tetraplegia or paraplegia)
- Immunosuppression (e.g. due to underlying disease or drug regime)
- Haematologic malignancy
- Severe coagulopathy at time of inclusion

#### Definition/conditions

##### Inclusion criteria

*Inclusion within the first 48 h after ICU admission.* All patients admitted to the ICU fulfilling the inclusion criteria will be screened for eligibility. Since the study aims to assess beneficial effects of early enteral nutrition, patients not screened within 48 h after ICU admission will not be included in the trial.

*Adult patients (aged ≥ 18 years).* We will only include adult patients in our study.

*Expected enteral feeding of at least 5 days and expected ICU stay of at least 5 days.* For surveillance and comparison of the experimental and control groups, enteral nutrition should be administered for a minimum of 5 days. If the patient leaves the ICU between observation days 5 and 7, the muscle biopsy and the ultrasound measurement will be made at the time of discharge.

##### Exclusion criteria

*Start of enteral nutrition before ICU admission or study inclusion*. To provide the same baseline, patients will only be included whenever they did not receive enteral nutrition before study inclusion.

*Pregnant or breast feeding women*. A negative pregnancy test (i.e. beta-hCG in urine or serum) will be necessary prior to study inclusion in women aged < 45 years.

*Clinically significant acute or chronic kidney failure with GFR < 20 ml/min.* Schwartz et al. [34] concluded that although significant restrictions in protein intake may not be necessary for critically ill patients with acute kidney injury, excessive protein provision in these patients could have a negative effect on renal function and lead to azotaemia. For safety reasons, the GFR value is intentionally set low to exclude patients with severely restricted kidney function.

*BMI ≤ 18 or ≥ 35 kg/m*^*2*^. Patients with underweight or obesity will be excluded from the trial because of altered muscle mass, pre-existing endocrine dysfunction or diverging energy or protein requirements.

*Intestinal perforation, peritonitis, intestinal fistula, necrosis or other contraindication to enteral nutrition.* Gastrointestinal conditions will be excluded to prevent adverse effects of enteral feeding and to decrease the number of patients not completing the study protocol.

*Noradrenaline ≥ 0.5 μg/kg BW/min.* Noradrenaline leads to decreased blood circulation in the muscles. Therefore, analysis of muscle biopsies might be biassed and incision healing negatively affected due to local decrease in blood circulation.

*Inherited and chronic skeletal muscle disorder (e.g. motor neuron disease).* Because of altered muscle metabolism and lower total muscle mass, the analysis of muscle biopsies might be biassed if these patients are included in the study.

*Paralysis (e.g. hemiplegia, tetraplegia and paraplegia).* Because of altered muscle metabolism and lower total muscle mass.

*Immunosuppression (e.g. due to underlying disease or drug regime)*. Patients with immunosuppression suffer from increased risk of infection due to muscle biopsy. Additionally, corticosteroids are known for their influence on muscle breakdown.

*Haematologic malignancy.* Haematologic malignancies might negatively affect blood coagulation and lead to increased bleeding after muscle biopsy.

*Severe coagulopathy at time of inclusion.* Because of increased intramuscular bleeding and haematoma after the muscle biopsy.

### Patient informed consent

Since the study includes a particular and vulnerable population, often not being able to give consent themselves due to, for example, sedation, there is a defined approach to obtain informed consent.

Whenever a potential study participant will not be capable to give his/her consent for the study themselves because of intubation or general restricted constitution, an independent auditing physician, acting as the patient’s representative, will declare the patient’s suitability for trial participation in the patient’s name. The signed document of the independent physician will be the prevailing condition for including the patient in the study. As soon as possible, a member of the study team will talk to the patient’s next of kin or preferably to the patient him/herself and explain the nature of the study, its purpose, the procedures involved, the expected duration, the potential benefits and risks and any discomfort it may entail. The patient or his/her next of kin will be informed that the participation in the study is voluntary and withdrawal from the study is possible at any time and that withdrawal of consent will not affect the subsequent medical assistance and treatment. The patient’s next of kin will be advised to decide according to the patient’s presumed will. If possible, formal consent from a participant’s next of kin, using the approved consent form, must be obtained before the participant is subjected to any study procedure. As soon as feasible, written informed consent from the patient him/herself must be obtained. Wherever possible, the participant or the next of kin must be informed that authorised institutions other than their treating physicians may examine the medical record.

A participant information sheet will be given to all consenting participants and to consenting next of kin deciding in their relative’s behalf. This information sheet describes the study and provides sufficient information for the participant and his/her next of kin to make an informed decision about the participation in the study. There will be a consent form for the patient and a secondary form for the patient’s next of kin. The patient and next of kin information sheet and the consent forms have been submitted to the competent ethics committee for revision and have been approved.

If possible, the patient or the next of kin should read and consider the statement before signing and dating the informed consent form and will receive a copy of the signed document. The consent form signed by the patient and/or his/her next of kin must also be signed and dated by a member of the study team. The original signed form will be stored along with the study-specific documents.

Whenever the participant or the patient’s next of kin refuses or withdraws his/her consent, the patient will immediately be excluded from any study interventions.

### Model consent form and other related documentation given to participants and authorised surrogates

These documents are available from the corresponding author on request.

### Additional consent provisions for collection and use of participant data and biological specimens

As stated previously, in the consent form, participants will be asked if they agree to use of their data should they choose to withdraw from the trial. Participants will also be asked for permission for the research team to share relevant data with people from the Universities taking part in the research or from regulatory authorities, where relevant. This trial involves collecting biological specimens for storage.

### Interventions

#### Explanation for the choice of comparators

Continuous enteral feeding represents current standard and will serve as the direct comparator to the intervention with intermittent feeding. Our trial will examine two different application protocols with high-protein enteral feeding.

#### Intervention description

In both the experimental (IF) and control (CF) groups, the high-protein (i.e. 10 g protein per 100 ml; 1.13 g leucine/100 ml) whey-based formula Fresubin© Intensive (Fresenius Kabi Schweiz AG) will be administered by nasogastric tube. Fresubin© Intensive is a specific enteral formula used in critically ill patients during acute phase to deliver a high amount of protein. A detailed list of ingredients can be found in Supplementary Table 1. Based on expert consensus, in the absence of indirect calorimetry (IC), we suggest that a published predictive equation or a simplistic weight-based equation (25–30 kcal/kg/day) be used to determine energy requirements. Dose and administration regime are indicated in Table 1 for the experimental group and in Table 2 for the control group.

**Table 1:** Intermittent feeding protocol (experimental group)

Time	06:00–06:30		10:30	11:00–11:30		15:30	16:00–16:30		20:30	21:00–21:30		01:30	
	Nutrition	Break	GRV	Nutrition	Break	GRV	Nutrition	Break	GRV	Nutrition	Break	GRV	Break
**Day 1**													
Protein dose^1^	0.1			0.2			0.4			0.4			
Flow rate^2^	50–90			100–180			200–360			200–360			
Total protein^3^	0.1			0.3			0.7			1.1			
**Day 2**													
Protein dose^1^	0.4			0.4			0.4			0.4			
Flow rate^2^	200–360			200–360			200–360			200–360			
Total protein^3^	1.4			1.6			1.6			1.6			

*GRV* gastric residual volume

^1^[g/kgBW/30 min], 4 nutrition cycles/24 h: each with 30 min nutrition followed by a 4-h break and 30 min GRV measure, night time break of 4 h. Total time of feeding, 2 h; GRV measurements, 2 h; break, 20 h

^2^[ml Fresubin© Intensive/30 min], depending on bodyweight

^3^Total of administered protein of the preceding 24 h [g/kgBW]

**Table 2:** Continuous feeding protocol (control group)

Time	06:00–11:00	11:00–12:00	12:00–17:00	17:00–18:00	18:00–23:00	23:00–00:00	00:00–05:00	05:00–06:00
	Nutrition	Break + GRV	Nutrition	Break + GRV	Nutrition	Break + GRV	Nutrition	Break + GRV
**Day 1**								
Protein dose^1^	0.05		0.1		0.2		0.3	
Flow rate^2^	5–9		10–18		20–36		30–54	
Total protein^3^	0.05		0.15		0.35		0.65	
**Day 2**								
Protein dose^1^	0.4		0.4		0.4		0.4	
Flow rate^2^	40–72		40–72		40–72		40–72	
Total protein^3^	1.0		1.3		1.5		1.6	

*GRV* gastric residual volume

^1^[g/kgBW/5 h], 4 nutrition cycles/24 h: each with 5 h nutrition followed by a 30-min break and 30 min GRV measure, no night time break. Total time of feeding, 20 h; GRV measurements, 2 h; break 2 h

^2^[ml Fresubin© Intensive/h], depending on bodyweight

^3^Total of administered protein of the preceding 24 h [g/kgBW]

The experimental group will receive a calculated amount of enteral nutrition over 30 min, whereas the control group will receive the same amount over 5 h. After a 4.5-h break in feeding in the experimental group and a 1-h break in feeding in the control group, the next feeding cycle will begin. The experimental group will have a 4-h break at night.

The experimental group will start with 0.1 g protein/kg BW and will receive 0.2 g protein/kg BW in the next cycle, which again will be doubled within the third cycle to 0.4 g protein/kg BW. The control group will start with 0.05 g protein/kg BW. With the second cycle, patients receive 0.1 g protein/kg BW with further increase by 0.1 g protein/kg BW with every cycle until the amount of 0.4 g protein/kg BW is achieved. The protein target will be reached at the cumulative dose of at least 1.5 g protein/kg BW/24 h over 4 consecutive feeding cycles.

For calculation of our protein targets of participants with BMI > 18 to < 30, the actual body weight will be used. For participants with a BMI of 30–34.9 kg/m^2^, the target protein amount will be matched for an ideal weight with a BMI of 25 kg/m^2^. Hence, indicated flow rates in Tables 1 and 2 are calculated based on protein targets which are prioritised to energy targets during the acute phase of ICU stay.

#### Criteria for discontinuing or modifying allocated interventions

Participants will be withdrawn from the study in case of the following:

- Death or rapid recovery/discharge
- On demand of the patient or his/her next of kin
- On demand of the treating physician or the study team (e.g. safety reasons, please see the “Adverse event reporting and harms” section)

#### Strategies to improve adherence to interventions

To improve adherence to intervention protocols and monitoring adherence, all study patients receive a bedside protocol with the study group, feeding cycles and calculated amount of nutrition, space for documentation of adverse events within every nutrition cycle. The study team fulfils daily controls and if needed reminds bedside nurses to document all the values needed for the study.

#### Relevant concomitant care permitted or prohibited during the trial

Implementing intermittent and continuous enteral nutrition with a protein-rich formula in critically ill patients will not require alteration to usual care pathways (including use of any medication), and these will continue for both trial arms.

#### Provisions for post-trial care

All participants will be monitored independent from the study according to ICU standards. Participants experiencing pulmonary aspiration during the study period will be followed up until hospital discharge. Apart from time to ICU discharge, potential complications of aspiration until hospital discharge—such as pneumonia, empyema, lung abscess, pleural effusion, sepsis and acute respiratory distress syndrome (ARDS)—will be registered.

#### Outcomes

##### Primary outcome measure

The primary outcome is the time in hours from the first study-specific administration of enteral nutrition until individual protein target quantity of at least 1.5 g protein/kg BW/24 h is reached. The daily protein target quantity is relative to the bodyweight and therefore varies among patients. According to the ASPEN guidelines for the ICU enteral nutrition [35], our target is 1.5 g protein/kg BW/24 h and should be reached in acute phase nutrition of critically ill patients.

##### Secondary outcome measures

The following secondary outcome measures will be assessed to detect differences among both feeding protocols:

- Evolution of values (i.e. the trend of the assessed parameters during the 7-day study period) for anabolic and catabolic pathways (ubiquitin-proteasome and autophagy) which control muscle metabolism as identified by proteomic analysis of vastus lateralis muscle biopsies prior to the start of nutrition and on day 7 in both study groups
- Quadriceps muscle area and diameter by ultrasound prior to start of nutrition and on day 7 in both study groups
- Amount of gastric residual volume (GRV) in millilitres per day and assessed after every nutrition cycle
- Number of events of diarrhoea, constipation, meteorism, nausea, regurgitation, vomiting and pulmonary aspiration per feeding cycle:
  - Acute diarrhoea over ≥ 3 unformed to liquid bowel movements within 24 h and with a total weight of > 250 g per day are present.
  - Constipation is diagnosed if the defecation is more difficult and takes place less than three times a week; in the study, constipation will be defined at ≥ 3 days without bowel movements, whereby investigators must influence the stool regulation with medication.
  - Amount of regurgitation in millilitres per day and assessed after every nutrition cycle.
- Assessment of glucose values (mmol/l) for assessment of effect on glucose metabolism
- Assessment of urea (mmol/l) once daily as a marker for amino acid degradation
- Assessment of IGF-1 (insulin-like growth factor 1) once daily due to its influence on the MPS pathway
- Assessment of Acute Physiology and Chronic Health Evaluation (APACHE II; for severity of disease) and Simplified Acute Physiology Score (SAPS II; for risk of mortality) upon study inclusion (baseline), and daily assessment of Sepsis-related Organ Failure Assessment (SOFA; for degree of organ dysfunction)
- Nutrition Risk in Critically Ill (NUTRIC) score including interleukin-6 upon study inclusion (baseline)

#### Participant timeline

The study period consists of screening and enrolment, allocation, observation time and closeout. Screening and enrolment will be performed within 48 h after ICU admission. Allocation is defined as the day of start of enteral feeding. Closeout is defined as the end of the seventh day of observation (or day of discharge from the ICU, if earlier than 7 days).

##### Study period

The study period is 7 days beginning with the initiation of enteral feeding.

##### Study schedule

**Fig. 1:**
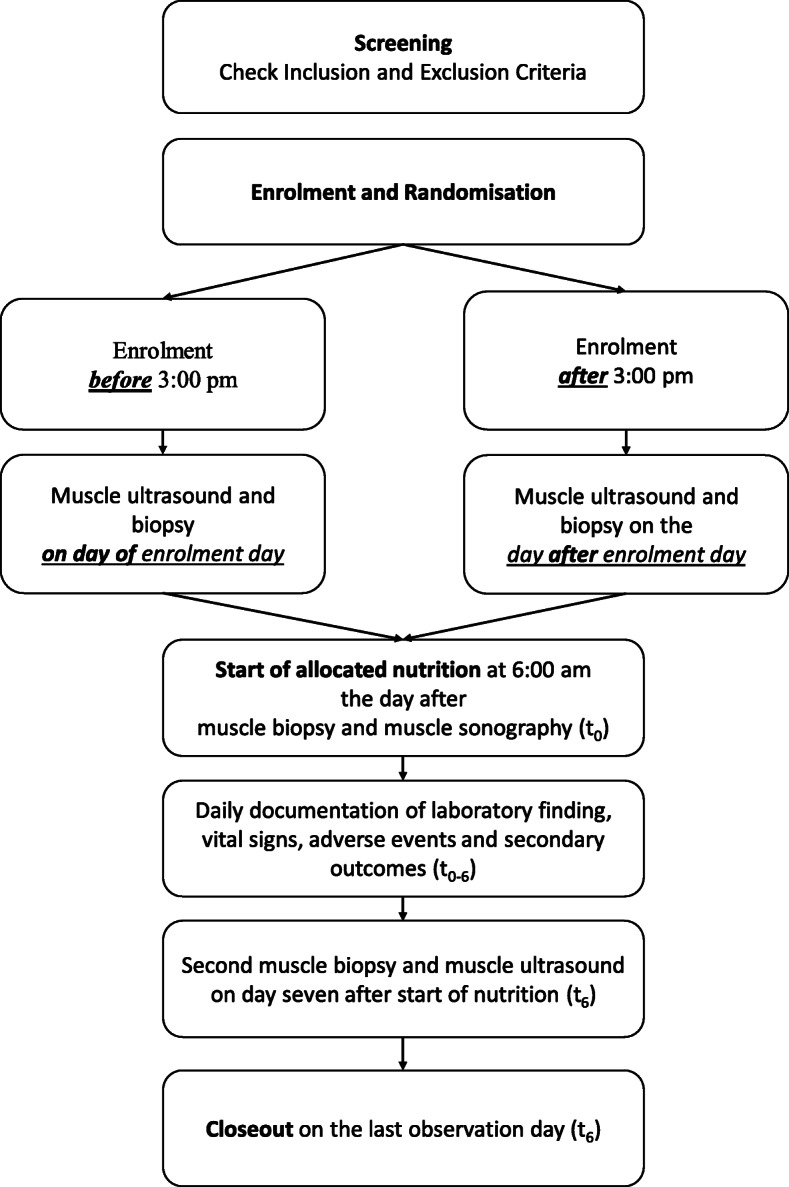
Study Flow Chart

Study flow is shown in Fig. 1 and Table 3.

**Table 3:** Study schedule

	Screening	Allocation	Observation time					Closeout
Time	t_−1_	t_0_	t_1_	t_2_	t_3_	t_4_	t_5_	t_6_
Day	0	1	2	3	4	5	6	7
Eligibility criteria	x							
Informed consent	x							
Randomisation	x							
Baseline data	x							
Muscle biopsy and ultrasound	x^1^							x
Start feeding		x^2^						
Vital parameters	x	x	x	x	x	x	x	x
Primary variables		x	x	x	x	x	x	x
Secondary variables^3^	x	x	x	x	x	x	x	x
SAEs		x	x	x	x	x	x	x

^1^Performance of muscle ultrasound and biopsy on inclusion day until 3:00 pm will allow enteral feeding starting the next day at 6:00 am. Whenever the study team is not able to perform both before 3:00 pm, these study procedures will be postponed to the next day and study feeding will start the day after at 6:00 am

^2^6:00 am

^3^Including laboratory parameters, documentation of side effects of enteral feeding and assessment of SOFA score

##### Day of inclusion (t_−1_)

We plan to perform the ultrasound and muscle biopsy on the day of study inclusion of eligible patients. Depending on time of ICU admittance, staff resources and opening hours of the Biobank storage, we will not always be able to do so. Therefore, if both the ultrasound and biopsy can be performed before 3:00 pm on the day of inclusion, enteral feeding will be initiated the next morning at 6:00 am. Otherwise, the procedures will be postponed to the next day and start of enteral feeding will be the following morning at 6:00 am. All standard laboratory parameters plus IGF-1 and interleukin-6 (for NUTRIC score) will be measured along with the morning routine laboratory of the next day. APACHE II, SAPS II and SOFA scores will be assessed within the first 24 h after ICU admission.

##### Day after muscle biopsy and ultrasound (t_0_)

Start of enteral feeding at 6:00 am is independent of the study group.

##### Days 1–7 of observation (t_0_–t_6_)

Laboratory parameters and SOFA score will be analysed daily.

##### Day 7 of observation (t_6_)

Muscle biopsy and ultrasound of the quadriceps muscle will be repeated. A closeout visit will be performed.

#### Sample size

Power analysis and sample size estimation were performed a priori using G*Power 3.1. We assumed a final 2-way repeated measures analysis of variance (ANOVA) and set a sample size to ensure at least 90% power (1 − *β* = 0.9) at a significance level of 1% (*α* = 0.01).

The effect size was estimated according to our feeding previous protocols (Tables 1 and 2): the average administered protein was 1.6 g protein/kg BW/24 h at 34 h in the experimental group and 1.3 g protein/kg BW/24 h at 36 h in the control group. The estimation of the mentioned daily protein targets in this study was based on a preceding prospective observational pilot feasibility study with a high-protein formula (manuscript currently under review). For practical reasons, the 2-h difference was deemed negligible. Protein targets have been chosen in accordance with the current guidelines for enteral nutrition [35–41]. For the time series analysis, a moderate correlation between consecutive time points (0.5, Hinkle et al. [42]) was assumed. The 20 time points were chosen according to the four scheduled measurements and calculation of applied amount of protein across each study day for a specified minimum follow-up of 5 days (see inclusion criteria).

Sixty patients are required to have a 90% chance of detecting an increase from 1.3 g protein/kg BW/24 h in the control group to 1.6 g protein/kg BW/24 h in the experimental group after 36 h.

#### Recruitment

The ICU study team will screen every patient with an expected need for enteral nutrition for a minimum of 5 days and a predicted ICU stay of at least 5 days within the first 48 h after ICU admission for study eligibility.

### Assignment of interventions: allocation

#### Sequence generation

Trial staff will have access 24/7 to the electronic case report form (eCRF) where patients are screened and randomised to one of the trial arms. Randomisation will be performed using electronic case report form (REDCap) (see the “Data management” section). A randomisation list will be established using computer-generated random numbers. We will use block randomisation, and patients will be stratified for neurotrauma.

#### Concealment mechanism

The allocation sequence will be uploaded upon study start. As soon as the eCRF is unlocked for real study data entry, there will be access for the study team to see the sequence.

#### Implementation

The allocation sequence will be generated in collaboration with the clinical trial unit. Recruitment and enrolment of participants will be executed by study team members. The assignment to interventions will automatically be performed by REDCap according to the allocation sequence implemented in the eCRF.

### Assignment of interventions: blinding

A blinding procedure for the treating medical team as well as the study team is not possible. However, the statistician who will analyse the final data will be blinded to the study group.

### Data collection and management

#### Plans for assessment and collection of outcomes

##### Assessment of primary outcome measure

The actual amount of protein intake within the last 24 h after every completed feeding administration cycle will be assessed. The actual amount of protein intake will be calculated according to the volume of the formula administered minus half of the recorded GRV. The decision to subtract half of GRV takes account of the fact that the recorded GRV will always consist of a mixture of nutrition formula and gastric juice. Results will be documented by the nurse responsible for the patient on a study-specific document and transferred to the eCRF, which will be accessible only to members of the study team. Assessment of primary and secondary outcome measures is outlined in Table 3.

##### Ultrasound measurement of quadriceps muscle prior to start of feeding and on day 7 in both study groups

The study team will perform the ultrasound measurements. The teaching for standardised quadriceps ultrasound will be provided and reviewed by the chief consultant of musculoskeletal diagnostics, Department of Radiology, University Hospital Basel.

Tillquist et al. recommend ultrasound imaging for determination of overall muscle mass in ICU patients due to excellent intra- and interrater reliability and the correlation between quadriceps muscle thickness (Q-MT) and overall muscle mass [43]. A linear probe of 5–10 MHz will be used. For comparability with other studies, the thickness of rectus femoris (RF) and vastus intermedius (VI) muscles and the total Q-MT will be measured. In addition, the cross-sectional area of rectus femoris (RF-CSA) will be measured. These parameters were found to be reliable and reproducible in former studies [33, 43–48]. The ultrasound measurements will be performed in the lower third in between the spina iliaca anterior superior and the upper border of the patella [43–45, 47, 49]. As recommended by Thomaes et al. [46], we will use minimal pressure and localise the probe perpendicular to the skin and at long axis of the thigh. Day-to-day consistency will be ensured using skin-compatible ink to mark the measurement sites. The examination will be repeated three times on each leg on both days (during screening (day − 1) and on closeout (day 7)). The mean of the three measurements of each leg will then be calculated and used for analysis.

##### GRV evolution and number of side effects of the enteral formula

GRV and adverse side effects caused by the enteral formula are listed among secondary outcome measures and will be assessed and documented by the nurse responsible for the study patient.

##### Glucose, urea and IGF-1 evolution over study period

Glucose, urea and IGF-1 will be measured together with daily routine blood analysis over the study period.

##### Assessment of study scores

The study team will assess and calculate standardised scores such as APACHE II, SAPS II and NUTRIC once within 24 h after ICU admission. The SOFA score will be assessed daily until closeout.

#### Plans to promote participant retention and complete follow-up

The defined eligibility criteria lead to participant retention due to inclusion of patients expected to stay at least 5 days in ICU.

In case of study withdrawal or early discharge within days 1–4 of the study, all data will be destroyed, and the patient will be replaced. Except for patients with early discharge to the general ward (e.g. before study day 5), the study team will attempt to continue the study: all study procedures will be continued including allocated feeding protocol, study laboratory and the ultrasound and muscle biopsy on day 7 of the study. If there is no possibility to continue enteral feeding on general ward, the patient will be replaced.

If the patient is discharged on day 5 or 6 of the study, we will perform the ultrasound and muscle biopsy on the day of discharge and accept missing data of the residual day(s).

If the patient is withdrawn from the study within days 5–7 of the study, the patient or his next of kin will be asked for use of the data already recorded. If applicable, we will also ask for use of regular data registered thereafter in the ICU setting.

#### Data management

Study data will be collected and managed using REDCap electronic data capture tools hosted at University Hospital Basel [50]. REDCap is a secure, web-based application designed to support data capture for research studies. REDCap provides (1) an intuitive interface for validated data entry, (2) audit trails for tracking data manipulation and export procedures, (3) automated export procedures for seamless data downloads to common statistical packages and (4) procedures for importing data from external sources.

All data, including demographic data, visit dates, randomisation number and serious adverse events (SAEs), related to this study will be kept within the trial master file (TMF) and the REDCap eCRF. A unique patient study code will be assigned to every screened patient consisting of the prefix PBN (Pro BoNo) plus consecutive numbering for every included patient (e.g. PBN-1). This code will be used to identify all study-specific data (e.g. ultrasound images, blood parameters). The trial will be terminated after collecting data from 30 patients in each group. The signed informed consent forms will be kept in the investigator site file (ISF). To minimise bias, we will set range checks for data values within the eCRF. Data will be archived dually on an external hard disk and on our in-house server system with a backup every 24 h. The hard disk and all physical study data will be stored in a designated place on our ICU at the University Hospital Basel for a minimum of 10 years after study termination or in case of premature termination of this study.

SAEs will be documented in the REDCap eCRF and on paper documents.

Only the study team will have access to study-related data. In case of a patient’s ex post denial of study participation, the data collected will not be used for publication in the present trial or in future trials. In such cases, the data will be destroyed.

#### Confidentiality

Except for the identification log and the informed consent, no study documentation will disclose the patient’s name. No personal identifying data will be registered within REDCap. Backtracking of personal data will only be possible using the identification log securely stored in the ISF. Access to the ISF will only be authorised to the study team and to staff performing monitoring visits.

#### Plans for collection, laboratory evaluation and storage of biological specimens for genetic or molecular analysis in this trial/future use

Proteomic analysis of muscle biopsies will be done prior to start of feeding and on day 7 in both study groups.

Study team members will be permitted to perform muscle biopsies after training with the head of the Neuromuscular Center of the University Hospital Basel. Biopsies of the vastus lateralis of the quadriceps muscle will be performed under local anaesthesia. A 14G disposable spring-loaded fine needle (Bard® Max-Core® Disposable Core Biopsy Instrument, Bard Biopsy, Arizona, USA) will be used. The biopsy will be taken at a penetration depth of 22 mm and maximal sample notch of 19 mm. The biopsies will be snap-frozen and stored at − 80 °C and processed histologically by the Department of Biomedicine of the University Hospital Basel. To evaluate the effect of IF and CF on muscle protein synthesis, proteolysis via the ubiquitin-proteasome pathway, autophagy flux and activation of signalling pathways will be determined. Protein kinase B [1, 3, 8, 25, 51–53], insulin-like growth factor 1 receptor [3, 54], glycogen synthase kinase 3-beta [3, 8, 52], mammalian target of rapamycin (mTOR) [1, 3, 8, 25, 51–54], eukaryotic initiation factors 2 [8, 25] and 4 [1, 8, 25, 51–54] and 70-kDa ribosomal protein S6 kinase [1, 3, 8, 25, 51–54] of the muscle biopsies will be quantified, and their activation level (mainly phosphorylation) will be determined. For the catabolic pathway, forkhead box class O-3 [1, 3, 52, 54], muscle atrophy F-box [1, 3], muscle ring-finger protein-1 [1, 3, 52] and nuclear factor kappa-beta [1, 3] will be measured. For information about autophagy, ULK1 [54, 55], light-chain 3 [55, 56] and p62 [55, 56] will be analysed. For an overview of enzyme regulation, AMP-activated protein kinase [8, 25, 53–55] will be studied.

### Statistical methods

#### Statistical methods for primary and secondary outcomes

Detailed methodology for summaries and statistical analyses of the data collected in this study will be documented in a statistical analysis plan elaborated with the Clinical Trial Unit of the University Hospital Basel.

##### Hypothesis

We hypothesise that target quantity of protein of at least 1.5 g protein/kg BW/24 h is reached earlier (i.e. within 36 h) in patients with IF than in those with standard CF.

##### Datasets to be analysed, analysis populations

An intention-to-treat and per protocol analysis of ICU patients who meet all inclusion and no exclusion criteria will be performed.

##### Primary and secondary analysis

A 2-way repeated measures ANOVA with post hoc pairwise comparison using a Bonferroni correction will be used to test for differences in protein administration at each time point for the control and experimental groups. The same method will be used to test differences in all secondary outcomes between groups. Additional statistical modelling using generalised linear models will be performed to inspect relationships between baseline patient characteristics, and primary and secondary outcomes.

All analyses on primary and secondary endpoints will be calculated by the study team in close collaboration with the Clinical Trial Unit of the University Hospital Basel.

#### Interim analyses

Interim analyses will be performed by the monitoring team. Data entry into the eCRF will be checked daily for completeness by the study team. The source data/documents will be accessible to monitors, and study-related questions will be answered during possible monitoring visits.

The sponsor-investigator may terminate the study prematurely in the following cases:

- Ethical concerns
- Insufficient participant recruitment
- When the safety of the participants is in question or at risk
- Alterations in accepted clinical practice that make the continuation of a clinical trial unwise (e.g. evidence against early enteral nutrition)
- Early evidence of benefit or harm of the experimental intervention

The trial will be stopped immediately. The participants and/or next of kin will be notified about the termination of the study. All collected associated data of the participants will be used for a preliminary analysis. This includes all associated muscle biopsies, ultrasound images and all values reported in the eCRF.

#### Methods for additional analyses (e.g. subgroup analyses)

We stratified for patients suffering from neurotrauma, which will represent a subgroup for analysis.

#### Methods in analysis to handle protocol non-adherence and any statistical methods to handle missing data

Despite the low number of participants, no considerable amount of missing data is expected due to the close observation of patients on the ICU in addition to our stipulations for replacing withdrawn patients according to the protocol. As described under the “Patient informed consent” section, the patients and their next of kin will have the option to agree to study participation whilst rejecting performance of muscle biopsies. Nevertheless, the study team is interested in obtaining as much study data as possible but will accept missing data on secondary outcomes due to lack of consent for the muscle biopsy. Patients with missing muscle biopsies will not be replaced. No imputation of missing data will be performed for statistical analysis.

#### Plans to give access to the full protocol, participant-level data and statistical code

The sponsor has full access to all data in the study and takes responsibility for the integrity of the data and the accuracy of the data analysis. The datasets analysed during the current study are available from the sponsor or the corresponding author on reasonable request.

### Oversight and monitoring

#### Composition of the coordinating centre and trial steering committee

The trial will be organised and managed by the research team of the intensive care unit in collaboration with the ICU staff. Muscle ultrasonography and biopsies of the quadriceps will be performed by the study team. Assessment of the other secondary outcomes will be supervised by advanced nurse practitioners. Monitoring will be provided by an experienced study nurse. REDCap data collection system will be established by the study team together with the REDCap collaborator at the University Hospital Basel. A member of the study team will conduct daily monitoring of eCRF performance. The statistical research plan and statistical analysis will be supervised by the Clinical Trial Unit, University Hospital Basel.

Co-enrolment of study participants in other clinical trials will be allowed but will have to be discussed among the competing research teams prior to randomisation.

#### Composition of the data monitoring committee, its role and reporting structure

A study nurse of the University Hospital of Basel will be responsible for study monitoring. Monitoring visits at the investigator’s site are planned according to the pre-defined monitoring plan approved by the local ethics committee. Monitoring will commence with a trial initiation visit (TIV), followed by regular monitoring visits within time frames that will have to be determined according to recruitment progress. Monitoring visits will be documented, and information stored within the ISF. Monitoring will be independent from sponsor and competing interests.

The source data/documents are accessible to monitors, the competent ethics committee and other involved competent authorities, and questions are answered during possible monitoring.

#### Adverse event reporting and harms

Since the utilised enteral nutrition is the standard formula for the initial phase in ICU patients at the University Hospital Basel, no severe adverse events relating to the components of this formula are expected after careful enrolment following the specified exclusion criteria. All study patients will be monitored with respect to any possible adverse events related to the administration of enteral nutrition. An accountability log for the nutrition formula will be provided for each patient with the batch number of the administered bags.

Fresubin© Intensive comes under risk category A in our trial. It is a medicinal product authorised in Switzerland, and its use in this study is in accordance with the prescribing information: critically ill ICU patients with increased need for protein. Possible side effects of intermittent feeding in the intervention group are expected to be similar to the familiar side effects (e.g. meteorism, diarrhoea and constipation) of continuous feeding. The aspiration risk will be limited due to frequent controls of GRV after every feeding cycle. The enteral feeding will be stopped immediately in case any adverse events, including GRV > 300 ml, vomiting, regurgitation or aspiration, occur. Feeding will be restarted at the next planned cycle at the highest dose previously tolerated. If a patient of the experimental group does not tolerate the administered dose of nutrition over three consecutive feeding cycles (despite common prokinetics as 100 mg erythromycin and 10 mg metoclopramide, whenever no contraindication is applicable), he or she will be crossed-over to the standard continuous feeding group. If a patient of the CF group does not tolerate the administered dose of nutrition over three consecutive cycles, the treating physician will discuss how to proceed together with the study team.

For monitoring of hypo/hyperglycaemia, we will determine the blood glucose with an interval of 3 h maximum and if applicable administer insulin according to our standardised ICU protocol considering the state of health and presence of diabetes mellitus.

The study will be carried out in accordance with the protocol and with policies detailed in the current version of the Declaration of Helsinki, the guidelines of Good Clinical Practice (GCP) issued by International Conference on Harmonisation of Technical Requirements for Registration of Pharmaceuticals for Human Use (ICH), Swiss law and Swiss regulatory authority’s requirements. The competent ethics committee (CEC) and regulatory authorities will receive annual safety and interim reports and will be informed about study stop/termination in agreement with local requirements.

##### Adverse events

According to the Swiss regulations for clinical trials in human research, it is not mandatory for clinical trials in risk category A to document adverse events. For safety reasons, side effects of enteral feeding as mentioned in the secondary outcomes will be documented during the course of this study.

##### Serious adverse events

Many ICU patients suffer from various adverse events that are life-threatening and/or prolong existing hospitalisation. Therefore, we will only document and report pulmonary aspiration (defined as evidence of nutrition formula within the tracheo-bronchial branches) or death of patients as SAE.

The occurrence of abovementioned SAEs will be assessed during every ICU shift. The sponsor must report all changes in research activity and unanticipated problems to the responsible ethics committee. Likewise, all SAEs must be reported within 7 days if fatal, otherwise within 15 days. The sponsor will provide an annual safety report.

#### Ethical justification

Due to impaired level of consciousness on the ICU, some patients may not be able to give their consent on their own. As described above, we will seek consent from the patient’s next of kin and/or if feasible the patient’s own agreement for study participation as soon as possible. We will only include patients who fulfil all inclusion criteria and no exclusion criteria to avoid adverse events and complications due to study interventions. Muscle weakness in critically ill ICU patients is a well-known problem that is associated with long-term morbidity and mortality. CF in ICU patients is currently recommended despite inherent disadvantages such as patients not reaching their protein target quantity or loss of pulsatile hormone secretion [57]. As such, IF is speculated to enable more physiological nutrition administration in these patients. With this study, we will contribute important data for discussion about the optimal form of enteral feeding schedule for critically ill patients.

#### Frequency and plans for auditing trial conduct

To assure correct trial conduct, the study team will discuss any insecurities and problems with monitor, especially in the beginning of the trial. Upon the TIV, the monitors will discuss the protocol in detail and identify and clarify any areas of weakness. Moreover, monitors or experienced members of the study team will organise a tutorial on the web-based data entry system and will support the practice of correct data entry. Both monitor and experienced study team members will audit the overall quality and completeness of the data and provide adequacy of due to close examination to confirm that the clinical centre has complied with the requirements of the protocol. The monitors will verify that all adverse events were documented in the correct format and that they are consistent with protocol definition. Both monitor and study team leaders will review documents and entered data for completion and adequacy. If a problem is identified during the visit (i.e. missing study documents or inadequate/missing data), the monitor and study team leaders will assist the site in resolving the issues.

#### Plans for communicating important protocol amendments to relevant parties (e.g. trial participants, ethical committees)

If for whatever reason there are substantial deviations of the analysis as outlined in these sections, the protocol will be amended.

### Dissemination plans

Study results will contribute to improving nutrition for ICU patients. During the ongoing study and until publication, there will be no public access to our data. We plan to publish the data in a major peer-reviewed clinical journal. A public description of the study in German is available on the Swiss National Clinical Trials Portal (SNCTP).

### Insurance

Insurance will be provided through the liability insurance of the University Hospital Basel.

## Discussion

### Trial rationale

We hypothesise that the target quantity of protein is reached earlier (within 36 h) with IF compared to CF. The results of this study may provide important data to define the future standard of enteral feeding protocols for the critically ill. This may lead to reduced long-term morbidity and mortality.

### Population

We will include patients expected to need enteral nutrition for at least 5 days and have an ICU stay of at least 5 days within 24 h after ICU admission.

### Intervention

In healthy, multiple studies show that intermittent eating not only is more physiological but also leads to increased protein synthesis. Therefore, intermittent eating leads to less degradation of skeletal muscles and provides pulsatile secretion of important hormones for metabolic function. In our trial, we aim to confirm the superiority of intermittent over continuous feeding concerning faster supply of protein via the enteral route in critically ill patients. The most common side effects of both enteral feeding regimens are meteorism, diarrhoea and constipation. Pulmonary aspiration would represent the most severe adverse event. Study data suggest that both forms of enteral feeding administration entail comparable risk of side effects when intermittent feeding is administered over 30 min.

### Outcomes

Based on evidence that intermittently fed patients may reach their protein target faster than continuously fed patients, we chose to evaluate the time (in hours) until patients reach the protein target of 1.5 g protein/kg BW/24 h to be able to calculate a significant reduction and thereby verify our hypothesis. Secondary outcomes will be used to compare tolerance of each enteral feeding regimen.

### Sample size

Sample size was estimated to prove the superiority of IF compared to CF regarding the time (in hours) to reach the protein target of 1.5 g protein/kg BW/24 h.

### Perspective

The Basel Pro BoNo study aims to generate new data on the effect of intermittent enteral feeding of critically ill patients on muscle wasting. Important topics such as when to start enteral nutrition in ICU patients and what protein amount should be targeted will lead to crucial discussions in the future. Additionally, the difference in required protein amounts in young versus elderly patients will have to be considered. In the end, nutrition only serves as one major and essential part in the approach of optimising muscle preservation in ICU patients. Improvement in reducing stress and immobilisation when treating ICU patients is indispensable.

## Trial status

The Ethics Committee of Northwestern and Central Switzerland (EKNZ 2018-00259 in April 2018) granted approval of this study in April 2018. The first patient was recruited in March 2019. Estimated study completion will be February 2021.

**Table:** 

Year	Procedure
2018	• Approval from competent ethics committee • Trial registration • Funding application • Establishment eCRF • Development of monitoring plan • Medical stuff study training
March 2019–February 2021	• Inclusion of 60 patients • Annual safety reports
2021	• Data analysis • Writing and submission of manuscript for publication

## Supplementary information

**Additional file 1: Supplementary Table 1.** Detailed information on Fresubin Intensive® enteral formula.

## Data Availability

The final trial dataset will be accessible to the sponsor and the study team members. The sponsor also holds the authorization over contractual agreements that limit such access for investigators.

## Abbreviations

AE: Adverse event
APACHE: Acute Physiology, Age, Chronic Health Evaluation
ARDS: Acute respiratory distress syndrome
BMI: Body mass index
BW: Bodyweight
CEC: Competent ethics committee
CF: Continuous feeding
eCRF: Electronic case report form
EKNZ: Ethics Committee of Northwestern and Central Switzerland
GCP: Good Clinical Practice
GFR: Glomerular filtration rate
GRV: Gastric residual volume
ICH: International Conference on Harmonisation
ICU: Intensive care unit
IF: Intermittent feeding
IGF-1: Insulin-like growth factor 1
ISF: Investigator site file
MD: Medical doctor
MPS: Muscle protein synthesis
NUTRIC: Nutrition Risk in Critically Ill
Pro BoNo: Protein Bolus Nutrition
Q-MT: Quadriceps muscle thickness
RF: Rectus femoris
RF-CSA: Rectus femoris cross-sectional area
SAE: Serious adverse event
SAPS: Simplified Acute Physiology Score
SNCTP: Swiss National Clinical Trials Portal
SOFA: Sepsis-related Organic Failure Assessment
TIV: Trial initiation visit
TMF: Trial master file
VI: Vastus intermedius
