# Supplementary material for: Protein delivery in intermittent and continuous enteral nutrition with a protein-rich formula in critically ill patients—a protocol for the prospective randomized controlled proof-of-concept Protein Bolus Nutrition (Pro BoNo) study
Source: Trials. 2020 Aug 25;21:740. doi: 10.1186/s13063-020-04635-1 (PMC7449093; doi:10.1186/s13063-020-04635-1)
Supplement: Supplementary file 1 — Additional file 1: Supplementary Table 1. Detailed information on Fresubin Intensive® enteral formula. [file 13063_2020_4635_MOESM1_ESM.docx]

**Supplementary**

Supplementary Table 1 **– Detailed information on Fresubin Intensive ® enteral formula.^1^**

| **100 ml contain:** | | |
| --- | --- | --- |
| Energy | 512 kJ | (= 122 kcal) |
| Fat (23 Energy%) | 3.2 | g |
| hereof saturated fatty acids | 1.66 | g |
| hereof medium-chain triglycerides | 1.28 | g |
| hereof monounsatureted fatty acids | 0.82 | g |
| hereof polyunsaturated fatty | 0.72 | g |
| Hereof EPA + DHA | 0.30 | g |
| Carbohydrate (42 Energy%) | 12.9 | g |
| hereof sugar | 8.0 | g |
| hereof lactose | ≤ 0.5 | g |
| Fibres (1 Energy%) | 0.64 | g |
| Proteins (33 Energy%) | 10.0 | g |
| Salt | 0.44 | g |
| Water | 80.5 | ml |
| Osmolarity | 600 | molmol/l |
| Osmolality | 740 | mosmol/kg H_2_O |
| **Minerals and Micro Nutritients** | |  |
| Sodium | 175 | mg |
| Potassium | 295 | mg |
| Chlorid | 160 | mg |
| Calcium | 105 | mg |
| Magnesium | 30 | mg |
| Phosphor | 70 | mg |
| Iron | 2.0 | mg |
| Zinc | 1.5 | mg |
| Copper | 230 | µg |
| Manganese | 0.48 | mg |
| Iodine | 22 | µg |
| Fluoride | 0.20 | mg |
| Chromium | 11 | µg |
| Molybdenum | 14 | µg |
| Selenium | 10.5 | µg |
| **Vitamins** | |  |
| Vitamin A | 130 | µg RE |
| Beta carotene | 300 | µg |
| Vitamin D_3_ | 2.0 | µg |
| Vitamin E | 3.0 | mg α-TE |
| Vitamin K_1_ | 9.0 | µg |
| Vitamin B_1_ | 0.23 | mg |
| Vitamin B_2_ | 0.24 | mg |
| Niacine | 2.4 | mg |
| Vitamin B_6_ | 0.27 | mg |
| Vitamin B_12_ | 0.5 | µg |
| Pantothenic acid | 0.9 | mg |
| Biotin | 6.8 | µg |
| Folic acid | 31.5 | µg |
| Vitamin C | 22 | mg |

1. Fresenius Kabi Deutschland GmbH. Fact Sheet Fresubin Intensive 2018 [updated May 2018; cited 2019 17.04.2019].01:[Available from: https://www.fresenius-kabi.com/de/documents/Datenblatt_Fresubin_ Intensive_2019.pdf.
